# Direct dotterising or angioplasty of acute stroke due to tandem atherosclerotic occlusions

**DOI:** 10.3389/fstro.2023.1163106

**Published:** 2023-05-25

**Authors:** Leonard L. L. Yeo, Davide Simonato, Pervinder Bhogal, Anil Gopinathan, Yang Cunli, Samuel W. Q. Ong, Mingxue Jing, Benjamin Y. Q. Tan, Ching-Hui Sia, Tom Jia, Giacomo Cester, Joseph-Domenico Gabrieli, Tommy Andersson

**Affiliations:** ^1^Division of Neurology, Department of Medicine, National University Health System and Yong Loo Lin School of Medicine, National University of Singapore, Singapore, Singapore; ^2^Department of Neuroradiology, Padua University Hospital, Padua, Italy; ^3^Department of Neuroradiology, St. Bartholomew's and The Royal London Hospital, London, United Kingdom; ^4^Division of Interventional Radiology, Department of Diagnostic Imaging, National University Hospital, Singapore and Yong Loo Lin School of Medicine, National University of Singapore, Singapore, Singapore; ^5^Department of Cardiology, National University Heart Centre Singapore, Yong Loo Lin School of Medicine, National University of Singapore, Singapore, Singapore; ^6^Department of Medical Imaging, AZ Groeninge, Kortrijk, Belgium; ^7^Department of Neuroradiology, Karolinska University Hospital, Stockholm, Sweden

**Keywords:** acute stroke, tandem occlusion, thrombectomy, carotid occlusion, treatment order

## Abstract

**Background:**

Tandem occlusions cause 10–15% of LVO acute ischemic strokes but are difficult to treat endovascularly and frequently excluded from clinical trials. The optimum endovascular method is still debated, however going directly through the carotid occlusion can speed up the procedure and reduce procedural risk by eliminating an exchange maneuver.

**Method:**

Using retrospective data from three centers, we compared treating atherosclerotic tandem occlusions using a 0.035'-guidewire and direct dotterisation or angioplasty with a peripheral vascular balloon suitable for the wire, vs. the usual technique of an 0.014'wire. We compared the successful recanalization (mTICI 2b-3) rates, 90 days' functional outcomes (mRS 0–2), and puncture-to-recanalization times between both procedures.

**Results:**

Forty-two consecutive patients with atherosclerotic tandem occlusions were included; 25 were treated with the 0.014'wire technique and 17 with the 0.035'-guidewire and direct dotterisation or angioplasty with a peripheral vascular balloon technique. The direct technique achieved a higher rate of successful recanalization (100 vs. 72%, *P* = 0.018), better functional outcome (88.4 vs. 48.0%, *P* = 0.044), and faster procedure times (mean 65.1 mins vs. 114.8 mins, *P* < 0.001). The number of attempts was similar between both groups (median 2 vs 3 attempts, *P* = 0.101). There was no significant difference in the complication rate between both groups (5.9 vs. 12.0%, *P* = 0.462).

**Conclusion:**

Compared to previous endovascular techniques for treating atherosclerotic tandem occlusions, the direct technique using standard 0.035' guidewires and dotterisation or a peripheral vascular balloon is significantly faster with better outcomes. However, this will require further external validation in larger cohorts.

## Introduction

Stroke is the main source of morbidity and dependence worldwide. While intracranial vascular occlusions are responsible for the majority of ischemic strokes, up to 10–15% of all ischemic strokes are associated with a stenosis or occlusion at the level of the internal carotid artery (Cremonesi et al., 2015). A tandem occlusion (TO), i.e. a thromboembolic obstruction in the intracranial cerebral vasculature in combination with an extracranial carotid artery occlusion or flow-limiting stenosis, can occur in up to one-sixth of ischemic stroke patients (Goyal et al., 2015). TOs tend to exhibit the worst prognosis of all the different types of acute ischemic stroke subtypes when treated with intravenous tPA alone. This is usually due to a large clot burden and reduced delivery of intravenous tPA to the clot if the collateral circulation is poor (del Zoppo et al., 2020). Endovascular treatment of TO during acute ischemic stroke has therefore gained widespread use. Despite this, the optimal endovascular procedure in acute TO generally remains unclear. The limited amount of evidence may be partially due to the exclusion of patients with an extracranial occlusion in several of the pivotal acute stroke thrombectomy studies (Nogueira et al., 2012; Saver et al., 2012, 2015; Pereira et al., 2013; Campbell et al., 2015). The thrombectomy trials which did include patients with TO, such as MR CLEAN and REVASCAT, demonstrated an incidence of TO between 17 and 32% (Berkhemer et al., 2015; Jovin et al., 2015). However, in these studies, the data are incomplete and inconclusive. For example, in the MR CLEAN study, only a minority (34.5%) of the patients with extracranial carotid disease were treated (Berkhemer et al., 2017).

This lack of evidence does not mean that TO strokes should not be treated, as successful reperfusion after LVO is an important predictor of a favorable outcome (Vanacker et al., 2014). Subgroup analyses of the ESCAPE and MR CLEAN studies have suggested that patients with TO should not be deprived of endovascular therapy; in contrast, outcomes may be better with early or concurrent treatment of the extracranial occlusion rather than later in a staged procedure (Berkhemer et al., 2017; Assis et al., 2018). Carotid stenting has also been suggested to have better outcomes and reperfusion rates than balloon angioplasty alone for tandem lesions (Zevallos et al., 2022).

There is controversy as to which is the optimal method of treating TO in acute stroke, i.e., is it better to initially (Cremonesi et al., 2015) by-pass the extracranial occlusion and remove the intracranial occlusion first before returning to tackle the extracranial stenosis, an approach which is termed the “retrograde”, or, is it preferable to (2) attempt primary recanalization of the extracranial occlusion first, before moving on to treat the intracranial occlusion, an approach termed “antegrade”? A recent meta-analysis on the subject showed that the retrograde approach may be associated with better outcomes. However, the exact technique was not defined (Zevallos et al., 2022). We studied a retrograde technique with direct treatment through the carotid stenosis that reduces the need for an exchange maneuver and therefore is less complicated. We evaluated if this method reduced procedure times and improved outcomes compared to the regular retrograde approach using a 0.014' guidewire.

## Methods

We included consecutive acute stroke patients from three stroke centers, with tandem occlusions treated with endovascular therapy but who did not qualify for intravenous thrombolysis due to being out of the time window. Patients had their NIHSS recorded as well as radiological variables such as the number of attempts, the devices used, and the antiplatelets used if any. Outcomes studied were the functional outcomes at 90 days (good outcomes were defined as mRS 0–2), successful recanalization (defined as mTICI grade 2b-3), the puncture-to-recanalization time, and the peri-procedural complication rate.

### Endovascular technique

**Figure 1:**
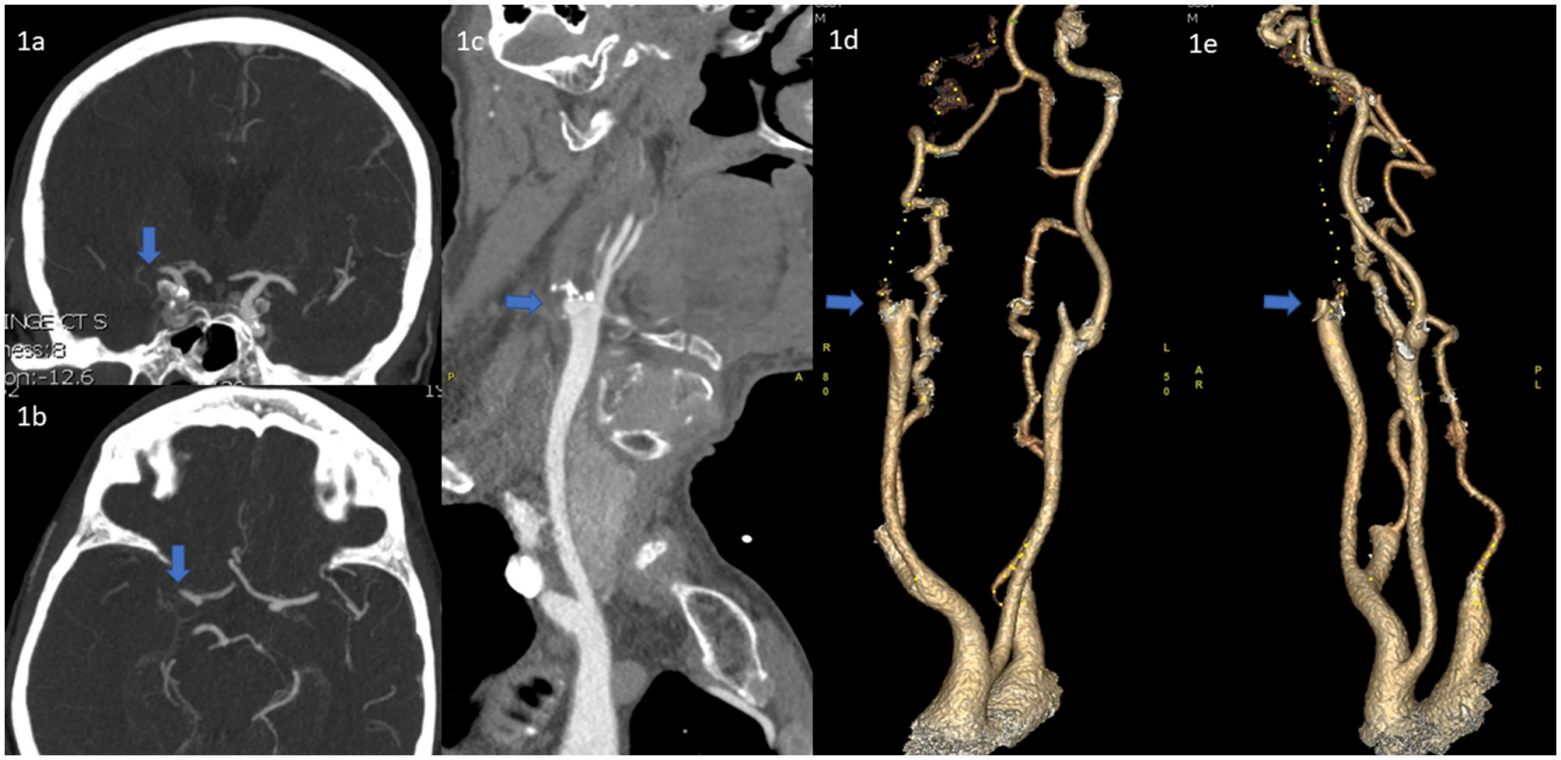
**(a, b)** CT-A images of the terminal ICA/proximal MCA occlusion. **(c)** The heavily calcified ICA occlusion at the bifurcation suggestive of an atherosclerotic lesion **(d, e)** 3d-reconstructions of the same ICA occlusion.

The choice of procedure, either the direct technique dotterisation or the usual technique of an 0.014'wire, was left up to the discretion of the individual interventionist. The direct technique dotterisation is briefly described here: An 80-cm long introducer sheath is placed in the upper mid-section of the common carotid artery (CCA) and an 8–9F balloon guide catheter (Flowgate, Merci - Stryker, Cello – Medtronic) or Neuron MAX guide catheter (Pneumbra) is positioned in the distal CCA or in the proximal ICA below the occlusion with a 120–125 cm 5F diagnostic catheter and a 260 cm 0.035-inch guidewire inside. The underlying etiology is then determined to be an atherosclerotic plaque by its appearance as well as by utilizing indirect evidence such as the age of the patient, location of the occlusion, and the presence of atherosclerotic changes elsewhere in the vasculature (Figures 1a–e).

The atherosclerotic ICA occlusion is carefully passed with the 260 cm 0.035-inch guidewire under flow arrest created by inflating the BGC in the proximal ICA or in the distal CCA as close as possible to the origin of the external carotid artery. The 0.035-guidewire tip is kept clearly visible distal to the occlusion at all times. The diagnostic catheter is then advanced past the occlusion. If the Neuron MAX is used it is then used to dotterise the occlusion with continuous aspiration; if a BGC is used, it is deflated and used to dotterise the occlusion with similar aspiration.

**Figure 2:**
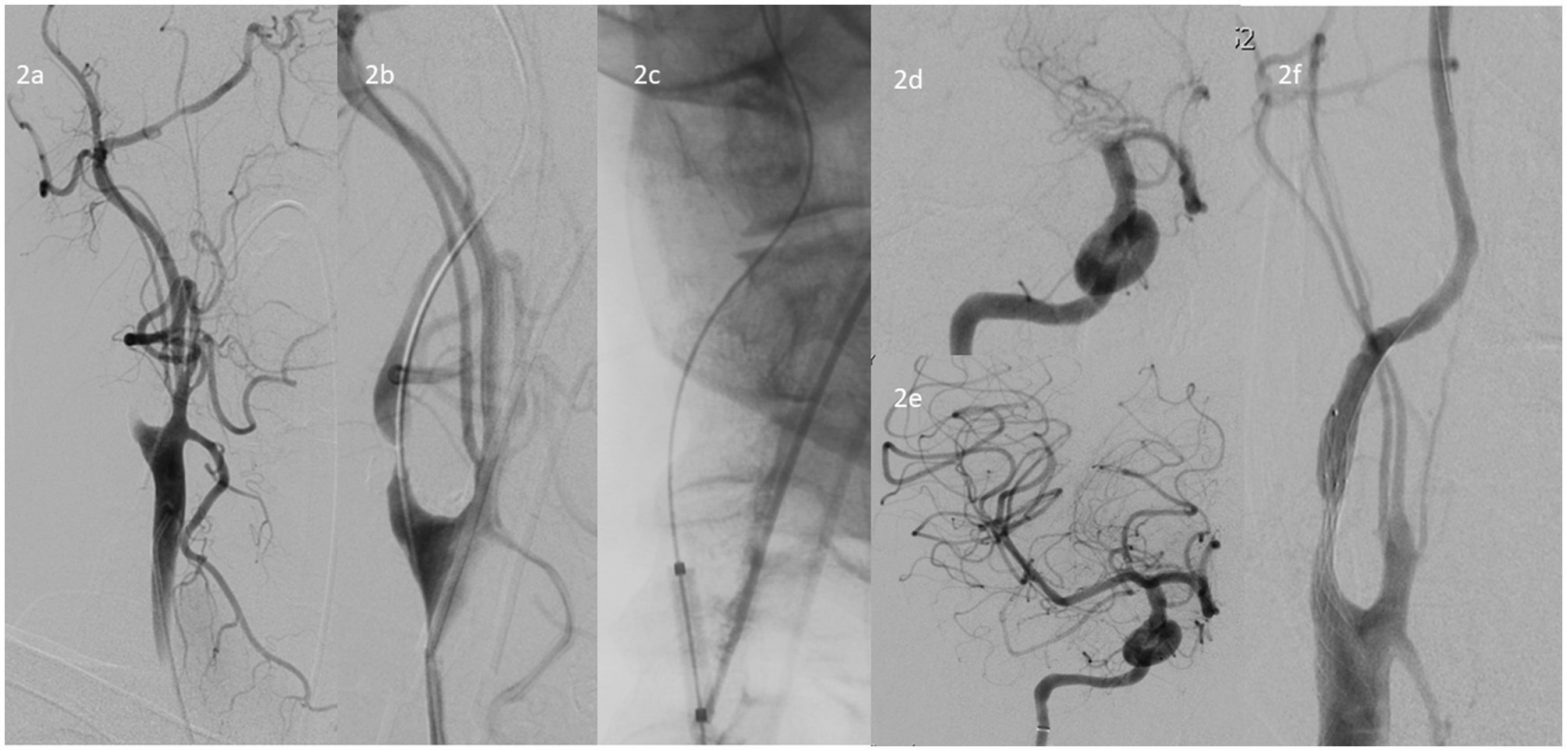
**(a)** DSA of the ICA occlusion at the bifurcation **(b)** Crossing the occlusion with a 260 cm 0.035-inch guidewire, **(c)** Angioplasty of the ICA occlusion with a 5 mm over-the-wire balloon, typically used for peripheral vessels, in this case a Passeo-35 5 × 20 mm balloon, **(d)** DSA showing the TICA/proximal MCA occlusion, **(e)** Post-thrombectomy DSA of the intracranial proximal MCA occlusions, **(f)** Subsequent stenting of the residual ICA stenosis with a stenting with Roadsaver 8 × 20 mm stent.

If the lesion cannot be passed, a 5 mm over-the-wire balloon, typically used for peripheral vessels (e.g., Passeo, Biotronic, or Mustang, Boston Scientific), is then advanced over the 0.035-guidewire and gentle angioplasty of the ICA-occlusion is performed (Figures 2a–c). If a BGC is used, this is done with the BGC inflated and the BGC-hub open. After aspiration in the BGC, it is then deflated and the now opened ICA-occlusion is first gently passed with the long 5F inner catheter and then subsequently dotterised with the BGC or Neuron MAX so that the tip is above the occlusion site. The 0.035-inch guidewire and the diagnostic catheter are removed and a microcatheter and microwire are advanced into the intracranial circulation. A typical thrombectomy with is then performed (Figures 2d, e) (Turk et al., 2014; Behme et al., 2015; Maus et al., 2018).

When reperfusion to the brain has been established, the BGC or Neuron MAX, which remains with the tip above the previous occlusion, is used to deploy a carotid stent. Because of the position of the BGC, this maneuver can be performed without the aid of a microwire. Post-dilatation tends not to be performed, as the indication for stenting in this patient group is to prevent re-occlusion, not to completely re-establish full blood flow in the carotid, and may cause “cheese grating” of the plaque through the stent with distal emboli. Finally, patients are given 500 mg of IV aspirin or GP2b/3a inhibitors.

## Results

Forty-two consecutive patients with atherosclerotic tandem ICA and MCA occlusions were included in this analysis; 25 were treated with the usual retrograde technique using the 0.014'wire technique to pass the occlusion, thus requiring an exchange maneuver, while 17 were treated with the direct 0.035' wire with dotterisation by the BGC or guide catheter technique. The mean age was 67 (SD 11.3) years old and 28 (57.1%) were male patients. The median NIHSS was 16 (range 3–30) and the median number of attempts was 2 (range 1–6). Good functional outcomes at 90 days were achieved by 55.8% of the patients and successful recanalization (mTICI 2b-3) was achieved by 83.4% of the cohort (Table 1).

**Table 1:** List of atherosclerotic tandem occlusion cases.

**S/N**	**Age**	**Sex**	**IV tPA**	**Method**	**Occlusion**	**NIHSS onset**	**mTICI**	**Attempts**	**PTR time (mins)**	**mRS at 90 days**	**Stenting**	**Antiplatelet**	**Complications**
1	50	Male	Yes	Old retrograde	ICA and M2	30	3	3	196	2	no	Aspirin + Plavix	Nil
2	89	Female	No	Old retrograde	ICA and M1	22	2a	6	115	4	no	Aspirin + Plavix	Nil
3	46	Male	Yes	Old retrograde	ICA and M1	12	0	3	143	4	no	Aspirin + Plavix	Nil
4	66	Female	Yes	Old retrograde	ICA and M1	10	3	3	120	3	no	Aspirin + Plavix	Nil
5	62	Male	No	Old retrograde	ICA and M1	14	2b	2	88	3	no	Aspirin + Plavix	Nil
6	71	Male	Yes	Old retrograde	ICA and M2	8	2b	2	100	1	no	Aspirin + Plavix	Nil
7	82	Female	Yes	Old retrograde	ICA and TICA	23	0	4	114	5	no	Aspirin + Plavix	Bleed
8	76	Male	No	Old retrograde	ICA and M1	15	2b	2	99	1	Stent acute	Reopro half dose	Nil
9	65	Male	Yes	Old retrograde	ICA and M1	14	2c	2	123	4	Stent acute	Reopro half dose	Nil
10	68	Male	Yes	Old retrograde	ICA and M1	14	3	1	101	2	Stent acute	Reopro half dose	Nil
13	68	Female	No	Old retrograde	ICA and TICA	11	2b	1	121	3	Stent acute	Reopro half dose	Nil
14	81	Male	Yes	Old retrograde	ICA and M1	12	2c	1	75	2	Stent acute	Reopro half dose	Nil
15	69	Female	No	Old retrograde	ICA and M1	10	2a	2	89	2	Stent acute	Reopro half dose	Nil
16	54	Female	No	Old retrograde	ICA and M1	18	3	1	78	1	Stent acute	Reopro half dose	Nil
17	86	Male	Yes	Old retrograde	ICA and M1	10	2c	1	206	6	Stent acute	Reopro half dose	Bleed
18	47	Female	No	Old retrograde	ICA and M1	19	2a	1	112	4	Stent acute	Reopro full dose	Nil
19	67	Male	Yes	Old retrograde	ICA and M1	19	2a	1	92	2	Stent acute	Reopro full dose	Nil
20	74	Female	No	Old retrograde	ICA and M1	19	2b	1	97	2	Stent acute	Reopro full dose	Nil
21	77	Male	Yes	Old retrograde	ICA and M1	20	2b	3	180	4	Stent acute	IV Aspirin 500mg	Nil
22	48	Male	Yes	Old retrograde	ICA and M1	16	2a	1	98	3	Stent acute	Integrillin full dose	Nil
23	75	Female	No	Old retrograde	ICA and M2	11	2b	3	100	0	Stent acute	ASA 500 mg e.v. + tirofiban	Intrastent occlusion
24	78	Male	No	Old retrograde	ICA and M1	22	2c	4	104	4	Stent acute	ASA 500 e.v.	Nil
25	74	Female	No	Old retrograde	ICA and M1	13	3	2	58	0	Stent acute	ASA 500 e.v.	Nil
26	65	Male	No	Old retrograde	ICA and M2	22	2c	2	210	0	Stent acute	ASA 500 e.v.	Nil
27	61	Female	No	Old retrograde	ICA and M1	16	3	3	52	2	Stent acute	ASA 250 e.v.	Nil
28	61	Male	Yes	Direct dotterisation	ICA and M1	18	3	3	80	3	no	Aspirin + Plavix	Bleed
29	72	Male	Yes	Direct dotterisation	ICA and M1	18	2b	3	59	2	Stent acute	tirofiban	Nil
30	57	Male	No	Direct dotterisation	ICA and M1	24	2b	2	72	1	Stent acute	Reopro half dose	Nil
31	83	Male	Yes	Direct dotterisation	ICA and M1	25	3	1	55	2	Stent acute	Integrillin full dose	Nil
32	46	Male	No	Direct dotterisation	ICAand TICA	18	3	4	55	4	No	ASA 500 e.v.	Nil
33	57	Male	No	Direct dotterisation	ICA and M1	16	2c	2	56	2	Stent acute	ASA 500 e.v.	Nil
34	63	Male	No	Direct dotterisation	ICA and M1	3	3	2	48	1	Stent acute	ASA 500 e.v.	Nil
35	70	Male	No	Direct dotterisation	ICA and M1	8	2c	6	81	1	Stent acute	ASA 500 e.v.	Nil
36	62	Male	No	Direct dotterisation	ICA and M1	18	2c	2	59	1	Stent acute	ASA 500 e.v.	Nil
37	72	Male	Yes	Direct dotterisation	ICA and M1	12	2c	3	82	0	Stent acute	ASA 500 e.v.	Nil
38	68	Male	No	Direct dotterisation	ICA, M2 and A3	21	3	2	69	4	Stent acute	ASA 500 e.v.	Nil
39	81	Female	Yes	Direct dotterisation	ICA and TICA	10	3	3	58	1	Stent acute	ASA 500 e.v.	Nil
40	74	Female	No	Direct dotterisation	ICA and TICA	18	2c	2	57	1	Stent acute	ASA 500 e.v.	Nil
41	61	Male	Yes	Direct dotterisation	ICA and TICA	15	2b	3	138	1	Stent acute	ASA 250 e.v. + tirofiban full dose	Nil
42	59	Male	Yes	Direct dotterisation	ICA and M1	8	2b	4	65	0	No	ASA 250 e.v.	Nil

IV tPA, intravenous tissue plasminogen activator; ICA, internal carotid artery; TICA, terminal internal carotid artery; NIHSS, national institute of health stroke scale; mTICI, modified Thrombolysis In Cerebral Infarction; PTR, puncture to reperfusion; mRS, modified Rankin scale; ASA, aspirin e.v.- endovascularly. Old retrograde approach- bypass extracranial occlusion with 0.014 wire and treat intracranial occlusion first, Direct dotterisation approach- bypass extracranial occlusion with 0.035 wire and dotterise with BGC or guide catheter, ICA- internal carotid artery, M1, M2 – Middle cerebral artery M1 or M2 segment, NIHSS- national institute of health stroke scale, mRS- modified Rankin Scale, PTR- puncture-to-reperfusion time, Antiplt- antiplatelet therapy.

On univariate analysis, the direct dotterisation technique was associated with shorter puncture-to-reperfusion time (mean 65.1 mins vs. 114.8 mins, *P* < 0.001), higher rate of successful recanalization (100 vs. 72%, *P* = 0.018), and better functional outcomes at 90 days (82.4 vs. 48.0%, P = 0.044). There was no significant difference in the complication rate (5.9 vs. 12.0%, *P* = 0.462) or the number of attempts between groups (median 3 vs. 2, *P* = 0.202) (Table 2). Two patients on whom the old retrograde technique was used had complications of symptomatic intracranial bleed and one patient had intra-procedural stent occlusion, while one patient with the direct dotterisation technique experienced a symptomatic intracranial bleed.

**Table 2:** A comparison of between the old retrograde technique and the direct retrograde technique dottering over the 0.035inch guidewire and using a peripheral balloon if needed.

	**Old retrograde method (*n* = 25)**	**New retrograde direct dotterisation method (*n* = 17)**	***P*** **-value**
Age	67.9 (12.0)	67.0 (9.9)	0.400
male	56.0%	82.3%	0.102
NIHSS (median, range)	15 (8–30)	16 (3–26)	0.473
Attempts (median, range)	2 (1–6)	3 (1–6)	0.101
Antiplatelet (IV 2b/3a)	52.0%	76.4.0%	0.074
Intravenous tPA	52.0 %	40.7%	0.500
mRS (0–2)	48.0%	82.4%	0.044
Puncture-to-reperfusion (mean, SD)	114.8 (42.2)	65.1 (23.2)	< 0.001
mTICI 2b-3	72%	100%	0.018
complications	12.0%	5.9%	0.462

The type of antiplatelet used was also not significantly associated with better functional outcomes (66.7 vs. 60%, *P* = 0.469) or increased complication rates (6.7 vs. 12.0%, *P* = 0.516). Two patients with the old retrograde technique had complications of symptomatic intracranial bleed and one patient had intra-procedural stent occlusion, while one patient with the direct dotterisation technique experienced a symptomatic intracranial bleed.

### Statistical procedures

We compared different variables between the two methods, where the Pearson Chi square test was used for categorical variables and the Mann–Whitney *U*-test was used for continuous variables. We performed statistical analysis using IBM SPSS statistics version 25.0 for Mac: SPSS Inc, USA.

## Discussion

We present a treatment strategy for acute ischemic stroke secondary to atherosclerotic TO. This approach uses 0.035-inch guidewires with a direct dotterisation using the guide or BGG, failing which a peripheral vascular balloon is used with less need for exchange maneuvers. This reduces the complexity of the procedure and shortens the procedure time with better recanalization rates and functional outcomes. Compared to the conventional technique, we demonstrate that this approach achieves better functional outcomes with a low complication rate.

Tandem occlusions can arise from a few different etiologies, however the two major causes for extracranial occlusions in TO are atherosclerosis and carotid dissection. A series of acute tandem occlusions suggest that about 60% are due to atherosclerotic plaques, while the remaining 40% are primarily due to dissection and a small number due to carotid webs (Papanagiotou et al., 2018).

Both causes differ significantly in the pathology of the occlusion as well as in the cerebrovascular hemodynamic circumstances. Irrespective of the cause of TO, since the introduction of the new generation of stent-retrievers, several studies demonstrated that thrombectomy of the intracranial clot and recanalization of the proximal lesion results in rates of favorable clinical outcome up to 68% (Brott et al., 2010; Berkhemer et al., 2017; Assis et al., 2018; Papanagiotou et al., 2018). In fact, sub-studies of these trials have shown that treatment of the carotid during acute stroke tended to have better outcomes when compared to patients with staged treatment or who underwent best medical therapy (Rubiera et al., 2006; Steglich-Arnholm et al., 2015; Berkhemer et al., 2017; Assis et al., 2018). It is the optimal endovascular approach for addressing the TO within the acute intervention period, which has recently been a subject of debate.

Most AIS-patients with TO are older and suffer from atherosclerotic disease with a ruptured plaque. This causes thrombus formation close to the carotid bifurcation which initially dislodges, sending an embolus to the intracranial circulation. The plaque may then remain severely stenosed or continue to occlude the carotid completely. Sending an embolus to the intracranial circulation requires that there is some flow left in the internal carotid and, in case of a TO, it is consequently likely that the carotid occlusion is acute and not chronic. This also means that the occlusion is typically able to penetrate and re-open. The other main reason for TO, especially in younger patients, is an acute dissection causing carotid occlusion or severe stenosis. This is usually located more distally, in the transition between the cervical and petrosal carotid sections.

While it can be difficult to differentiate atherosclerosis from a dissection, surrogate signs can be used such as the patient age, the presence of atherosclerotic plaques in other vessels, a tapering or “flame sign”, and a history of neck pain. It is important to make this distinction between dissection and atherosclerotic etiology because in most atherosclerotic plaques, the hemodynamic impairment is low due to its chronicity and the indication for acute stenting or angioplasty in the presence of hypoperfusion from this occlusion is low. Conversely, in a dissection, the occlusion is acute, and the brain has not had time to compensate for the hemodynamic impairment. The need for angioplasty and/or stenting to open the occlusion is therefore greater as there is a risk for hypoperfusion. Another factor is that the impaired autoregulation in a chronic atherosclerotic occlusion renders the parenchyma at a higher risk for reperfusion injury. In the case of a dissection, the acute occlusion means less risk of reperfusion injury.

Acute stenting of tandem lesions is less frequently performed for dissections than for atherosclerotic stenosis (Gory et al., 2017; Jacquin et al., 2019). This may be partly attributable to anatomical considerations (dissections have a tendency to be longer and more tortuous) as well as the more favorable natural evolution of carotid dissections, which have been described to heal on their own in up to 70% of the lesions, with subsequent recanalization of the occluded or stenosed vessel (Baracchini et al., 2010). Conversely, atherosclerotic tandem lesions are known to be more technically challenging with significantly longer procedural times, poorer recanalization rates, and increased risk of complications (Gliem et al., 2017).

Our proposed technique for atherosclerotic occlusions is largely an improvement on the proximal protection technique by using a BGC or a guide catheter over the 0.035-inch guidewire and using a balloon which is typically used for peripheral vascular angioplasty where necessary. This avoids the risk and delay of a guidewire exchange using the typical neurovascular balloons which fit on the 0.014-inch microwires.

The usual antegrade approach uses primary stenting to jail the extracranial stenotic atheromatous plaque, which should prevent showering of distal emboli (Lockau et al., 2015). A theoretical drawback of the antegrade approach is the procedural time used for carotid stent placement which delays the time to intracranial reperfusion, which might result in an increase of the final infarct volume (Lockau et al., 2015; Marnat et al., 2016). In our technique, the initial flow arrest by the BGC or dotterisation of the lesion and later bypassing the lesion has a similar effect, without the delay of the stent deployment. The BGC or guide catheter can then be used to deploy the carotid stent at the end of the procedure when there is no time pressure after the intracranial occlusion has been taken care of. One drawback of using the guide catheter to deploy the stent is that the operator is not able to withdraw the guide catheter to first re-assess the stenosis and see if it can be treated conservatively. Other centers have described a similar dilator-dotter method with identical rapid procedure times using a guide catheter (Woodward et al., 2016; Amuluru et al., 2020) however our study has used a balloon guide catheter and has a comparison to a control group.

Another important point to note is that the indication for stenting the carotid in an atherosclerotic TO is mainly to prevent re-embolization and not to completely normalize the blood-flow as this carotid was likely highly stenotic before the event. In case of dissection, conversely, the main indication of stenting is the prevention of a new thromboembolic event but also to avoid hypoperfusion as this carotid was most likely completely normal before the event and the hemodynamic risk is therefore a reality.

One shortcoming of the usual antegrade approach is that effective stent-retriever based thrombectomy techniques such as “Stent-retriever Assisted Vacuum-locked Extraction” (SAVE) or “Aspiration–Retriever Technique for Stroke” (ARTS) may not be able to be used after carotid stenting (Massari et al., 2016). This is because of potential entanglement between the struts of the stent-retriever and carotid stent during withdrawal when the guiding catheter could not be advanced through the carotid stent. This shortcoming will not be present in our technique.

### Limitations

This is a small retrospective cohort with all its inherent bias. The protocol was not uniformly enforced across all three centers and was largely left up to the individual to decide on the management strategy. The major limitation of the study is the lack of uniformity in the protocols by all participating centers. This may limit the generalizability of the results, at least until there is more data from improved designs. We did not have data on repeat imaging of the vessel to determine the re-stenosis or reocclusion rate, however we recorded the 90-day functional outcomes and a re-occlusion of the vessel would have presented with a repeat cerebral ischemic event that would have affected the functional outcomes. We were not able to control for the experience of operators or the bias that they may have had for one technique over the other.

## Conclusion

This is a strategy for treatment of atherosclerotic acute stroke tandem occlusions that takes advantage of BGCs/guide catheters and standard guidewires, while using peripheral vascular balloons if needed. It is potentially a faster technique with better outcomes. A larger cohort will be needed for validation.

## Data availability statement

The original contributions presented in the study are included in the article/supplementary material, further inquiries can be directed to the corresponding author.

## Ethics statement

The studies involving human participants were reviewed and approved by the DSRB. Written informed consent for participation was not required for this study in accordance with the national legislation and the institutional requirements.

## Author contributions

LY and TA provided direction, helped write the draft, provided cases and the images, and edited the manuscript. PB provided direction and edited the manuscript. AG, YC, GC, and J-DG provided cases and edited the manuscript. SO edited the manuscript. MJ, BT, GC, and C-HS contributed cases and edited the manuscript. DS helped write the draft, provided cases and the images, and edited the manuscript. All authors contributed to the article and approved the submitted version.
